# The sport experiences of blind or partially sighted people and strategies to support their participation in sport: A scoping review

**DOI:** 10.1177/02646196251330155

**Published:** 2025-05-15

**Authors:** Meredith K Wing, Julia Deuville, Alyssa C Grimes, Zachary Scanlan, Kelly Arbour-Nicitopoulos, Amy E Latimer-Cheung

**Affiliations:** Queen’s University, Canada; University of Toronto, Canada; Queen’s University, Canada

**Keywords:** Blind or partially sighted, coach, disability, quality participation, sport, support strategies

## Abstract

Programme leaders (PLs; e.g., coaches) are integral for fostering the quality participation (QP) of blind and partially sighted athletes. However, information about fostering QP for blind and partially sighted people is often inaccessible to PL. Informed by the Quality Parasport Participation Framework, we formulated this scoping review related to the sport participation of blind and partially sighted people and the strategies that support QP. Searching four databases, we screened 1245 studies and included 29 articles, generating insight related to study characteristics and the extent of in/direct references to the experiential elements of QP. After interpretive analysis, we constructed three principles to reconceptualize sport participation in relevant and affirming ways for blind and partially sighted athletes, as well as 33 foundational support strategies and 16 outcomes potentially associated with the QP. This project contributes to the visibility of blind and partially sighted athletes in the literature and in the Quality Parasport Participation Framework.

## Introduction

As a social institution, sport represents a dominant and influential aspect of society, with important implications for the health and wellbeing of individuals and communities (Spencer-Cavaliere et al., 2017). Often, sport is also positioned as an accessible social practice (MacDonald et al., 2012); however, numerous equity-owed groups, including blind and partially sighted people,^1^ face barriers across multiple life domains that diminish their opportunities to participate in sport (Martin Ginis et al., 2021; Ruin et al., 2023). For instance, a study exploring access to sport for blind and partially sighted people revealed that 62% of respondents were unable to participate in certain sports, often their favourite sports, due to a lack of adequate modifications (Maśliński et al., 2020). As such, many scholars in the literature have extensively discussed existing barriers and facilitators, while orienting their work towards fostering positive sport outcomes for blind and partially sighted people (e.g., greater rates of participation, improved wellbeing) (Meier et al., 2023) and moving beyond a simple focus on *quantity* of participation.

Recognizing that promoting attendance does not necessarily ensure positive sport experiences, scholars have called for broader conceptualizations of participation that focus on subjective experiences (Hammel et al., 2008; Imms et al., 2017). For example, Martin Ginis and colleagues (2017) described six experiential elements that make up subjective experiences during acute and in-the-moment (i.e., while engaged in an activity, not an outcome) sport participation: belongingness, autonomy, meaning, engagement, challenge, and mastery. Moreover, the authors suggested the construct of quality participation (QP), defined as repeated quality experiences (i.e., meaningful and positive) during which the needs of participants are satisfied across these six experiential elements (Martin Ginis et al., 2017). Based on this definition of QP, Evans and colleagues (2018) then outlined the Quality Parasport Participation Framework, herein referred to as the QP framework, to support further research centring the subjective experiences of athletes during acute participation; to describe the conditions that are conducive to quality experiences; and to encourage the development of quality sport programming for diverse participants.

While the QP framework has not yet been widely applied within the context of blind sport research or practice, there are many parallels between existent research and the QP framework. For example, Haegele and Maher (2023) centred the perspectives of blind and partially sighted people while integrating aspects of subjective and relational experiences in their (re)conception of inclusion. In particular, they emphasized the complex interplay between each autonomous person’s relational, perceptual, and embodied experiences of sport, as well as the influence of the group experiences constructed by those participating alongside them (Haegele & Maher, 2023). This complexity is similarly represented in the QP framework through the co-existing conditions of the physical, programme, and social environments that are shaped by each participant/collective and, in turn, can become strategies implemented within the collective to foster QP. Both Haegele and Maher’s (2023) conception of inclusion and the QP construct (Martin Ginis et al., 2017) characterize these experiences as dynamic and in flux, dependent on the unique individuals and collectives they form. Given these parallels, we believe that there are opportunities for scholars engaging these diverse approaches to further complement one another, particularly as we continue to explore possibilities for promoting the meaningful participation of blind and partially sighted people in current and future sport programmes.

Programme leaders (PLs) have a critical role in shaping the experiences of diverse disabled participants in mainstream and disability sport (Maher et al., 2023; Townsend et al., 2015). Despite their influence, Oldörp and colleagues (2024) emphasized that PLs are seldom adequately trained to support blind or partially sighted people. This is then compounded by the lack of available guidance regarding the quality experiences of blind or partially sighted athletes (Columna et al., 2019). To address the lack of guidance related to supporting diverse disabled people in sport more generally, the Canadian Disability Participation Project (CDPP, 2018) has developed and applied a process for creating evidence-informed resources including the Blueprint for Building Quality Participation in Sport for Children, Youth, and Adults with Disabilities (herein referred to as the blueprint). The rigorous and systematic blueprint development process centres around a review of existent literature to inform practical recommendations and has been applied numerous times since its creation. For example, the original blueprint is oriented towards the participation of people with physical disabilities and provides an overview of the development process, definitions of key terms, and 25 foundational strategies across the physical, programme, and social environments that can foster QP across the six experiential elements. Supported by the knowledge generated by Streatch and colleagues (2022) and Bruno and colleagues (2022), subsequent versions of the blueprint were created to specifically reflect the unique experiences and support needs of autistic children and children with intellectual and developmental disabilities (respectively), offering additional strategies that may be used to foster QP. To reflect the sport experiences and needs of other, diverse athletes, we sought to centre evidence describing the experiences and needs of blind or partially sighted people in an additional adaptation of the blueprint.

Guided by this goal, we conducted a scoping review to engage with the currently available literature pertaining to the sport experiences of blind or partially sighted people. More specifically, the purpose of the review was to conceptualize the positive, in-the-moment sport experiences of blind or partially sighted people and identify specific strategies that foster these experiences. To guide this review, we formulated the following research questions using the population/participants, concept, and context (PCC) approach suggested by Peters and colleagues (2020), as members of the Joanna Briggs Institute (JBI) Scoping Reviews Methodology Group:

*RQ1. What literature is currently available describing quality experiences of participation in sport among blind or partially sighted people?*
*RQ1a. Which experiential elements of QP are featured in the experiences described?*
*RQ2. How can the experiential elements of QP be fostered in sport for blind or partially sighted athletes (i.e., foundational strategies)?*
*RQ2a. Who, in what role(s), provides the support necessary to facilitate quality participation of this population?*
*RQ3. What outcomes are associated with QP in sport?*

## Methodology/methods

### Protocol and registration

The protocol for this project was formulated using the guidelines provided originally by Arksey and O’Malley (2005) and subsequently updated by Peters et al. (2020), as members of the JBI Scoping Reviews Methodology Group. On 22nd November 2023, this scoping review was registered on the Open Science Framework (OSF) platform (Wing et al., 2023).

### Search strategy

The librarian involved in the review, with extensive expertise in the field of human kinetics and related to disability and accessibility, identified the following four databases as relevant to the project: SportDiscus, Sports Medicine and Education Index, CINAHL, and APAPsycInfo. By searching all four of these databases, we captured literature from various disciplines to increase the breadth of our searches. Literature from all available years was included in the results; however, due to a lack of resources for translation, the searches were restricted to English language results.

The strategy employed in this review (see additional information in Supplemental Appendix A) encapsulated two main concepts: (a) disability and (b) sport. Keywords were chosen using the thesaurus and subject headings noted in the databases. Relevant terms, listed below, were identified and combined using the Boolean operators OR and AND; similar terms were combined using OR, while different concepts were combined using AND. Furthermore, each term was truncated and exploded where applicable. Due to the many uses of the word *blind* in various contexts apart from disability (e.g., experimental design, colloquial uses), results were limited by identifying commonly appearing irrelevant terms, then conducting searches to ensure their irrelevance, and combining them with the NOT operator. The results of these searches, conducted on 2 August 2024, were then uploaded to Covidence for deduplication and screening.

### Eligibility criteria

To be included, studies were originally required to pertain to the quality sport experiences, meaning positive or personally satisfying, of blind or partially sighted people of any age and/or contain strategies that foster quality sport experiences. Studies were not required to directly reference QP nor specifically discuss the six experiential elements. Reflecting the diversity across cultures and contexts, sport was defined in this review as structured activity that involves physical exertion, undertaken in pursuit of a particular goal (Eime et al., 2020). Studies were excluded for the following reasons: (a) pertained to the experiences of disabled people without a specific focus on blindness and partial sight; (b) related to an exercise, physical education, or physical activity context without any mention of sport; or (c) focused solely on quantitative or non-subjective outcomes such as performance or quantity of participation. As suggested by Arksey and O’Malley (2005), the review process was iterative, with regular reflection on our progress and any adaptations were made accordingly. In particular, we revisited our inclusion and exclusion criteria throughout the review process and, during data extraction, amended our original formulation of the criteria to also exclude studies solely representing parental perspectives, as well as studies that discussed athlete experiences in the context of inclusion without providing a further definition nor particular subjective experiences associated with the term. Moreover, due to the nature of the methodologies as secondary syntheses of knowledge, scoping and systematic reviews were also excluded; however, the references of relevant scoping and systematic reviews (e.g., Ball, Lieberman, Haibach-Beach, et al., 2022; Gamonales et al., 2018; Skelton et al., 2013; Tuakli-Wosornu et al., 2020) represented in the screened literature were explored to ensure our search was comprehensive.

### Study screening

**Figure 1.**
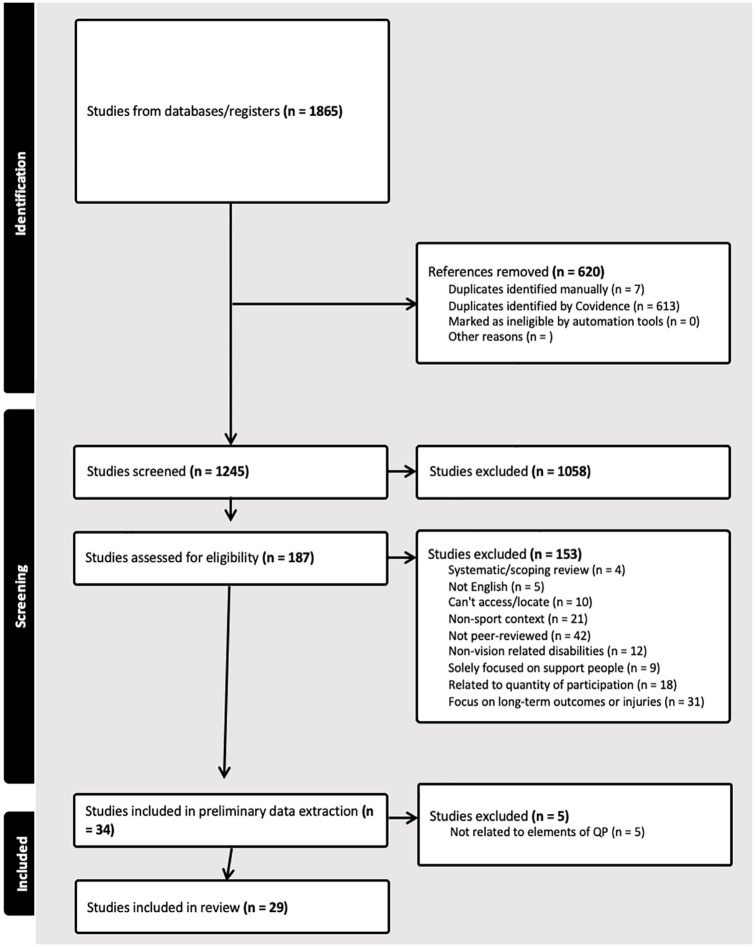
PRISMA flowchart.

The screening process involved three members of the research team (first and second authors and a student researcher), all using the same inclusion criteria (see Figure 1 for more information). To ensure that all reviewers were in agreement, these members of the research team met to discuss the review and, specifically, provide any necessary clarity on the inclusion criteria. Following deduplication, a total of 1245 studies were reviewed independently by the first and second authors against our inclusion criteria. Subsequently, 186 articles entered full-text screening – conducted independently by the first author and the student researcher. A total of 34 studies were included at the beginning of the data extraction process; however, after updating our inclusion/exclusion criteria, five studies were later excluded, for a total of 29 studies represented in this final iteration.

### Data extraction

From the 29 included articles (see Supplemental Appendix B for a full list), we extracted general study characteristics (see Table 1), QP experiential elements (see Table 2), strategies to foster QP (see Table 3), and outcomes related to QP (see Table 4). Of the 29 total articles, three studies recruited from or examined the same camp, Camp Abilities, although the studies were conducted in differing years. Given the interpretive nature of the scoping review, critical reflexive activities, including engagement with critical friends (Coghlan & Brydon-Miller, 2014) and personal reflection regarding one’s subjectivity, supported the review process and deepened the process of analysis, as outlined below.

**Table 1.** Summary of included articles.

Citation and country	Participant characteristics	Sport characteristics	Programme characteristics	Methodology
Barnett et al., 1993 USA	22 wrestlers, aged 13–18; 11 sighted athletes and 11 blind or partially sighted athletes	Wrestling	School-based team	Quantitative
Braga et al., 2015 USA	No participants directly engaged in study; pertains to blind and partially sighted students and students with cerebral palsy in mainstream education	Net/wall games	Higher education; inclusive	Programme review article
Brunes et al., 2017 Norway	2 blind or partially sighted individuals; 1 girl (12 years old) and 1 man (23 years old)	Multiple sports including track and field, martial arts, and swimming; team and individual sports	Community-based and integrated programmes	Qualitative
Columna et al., 2015 Guatemala	13 Latino parents of blind or partially sighted children (aged 5–18); 10 mothers and 3 fathers; recruited from Camp Abilities (sports camp for children and teens who are blind, visually impaired, and deafblind)	Structured and unstructured participation in sports, including extreme sports	Family integrated programmes, disability-specific camps, and integrated settings	Qualitative
Columna et al., 2017 USA	11 parents of blind or partially sighted children (aged 4–12); 10 mothers and 1 father	Structured and unstructured sport participation	Community-based programmes with inclusive and disability-specific models	Qualitative
de Schipper et al., 2017 USA	6 blind or partially sighted children; aged 10–12 years old; 3 girls and 3 boys	Mainstream and disability-specific sports	Community and school settings, inclusive and integrated programmes, and recreational and competitive levels	Qualitative
Esatbeyoglu et al., 2023 Türkiye	14 blind or partially sighted youth from Turkey; 8 boys and 6 girls; 13–18 years old	Capoeira (Afro-Brazilian traditional game and art form with elements of dance and martial arts)	3-week programme; disability-specific	Qualitative
Gombás & Gál, 2016 Hungary	140 blind or partially sighted people; 18–65 years old; men and women	Recreation sport; structured and unstructured participation	Community and school settings with inclusive and segregated models	Mixed methods
Goodwin et al., 2011 USA	13 blind or partially sighted youth; 9–15 years old; 7 girl and 6 boys; Asian, African America, Mexican American, and Euro American	Multi-sport camp	Residential sports camp (Camp Abilities); disability-specific	Qualitative
Green & Miyahara, 2007 New Zealand (Aotearoa)	6 blind or partially sighted older adults; aged 53–70; 3 men and 3 women	Shared experiences from participation in structured and unstructured sports	Disability-specific community programming (walking group)	Mixed methods
Haegele et al., 2014 USA	No participants directly engaged; pertains to blind and partially sighted students in mainstream education	Multi-sport	Camp Abilities; disability-specific	Practice report
Haegele et al., 2017 USA	4 Paralympic Goalball athletes; blind or partially sighted; White men; 22–37 years old	Many sport experiences, in addition to and supporting their participation in Goalball	Elite level, school (PE and extracurricular), and community programmes; mentioned Sport Education Camp	Qualitative
Hall et al., 2023 UK	10 White individuals aged 44–67; 5 blind or partially sighted runners and 5 sighted guides; 5 men and 5 women	Running; guided and independent; structured and unstructured participation	Programmes offered through community running clubs, as well as competitive races	Qualitative
Kirk & Haegele, 2021 USA	8 blind or partially sighted individuals; 21–34 years old; 5 women and 3 men; White, Asian, and African American	Structured and unstructured participation in sport; team and individual; mainstream and adapted sport	School and community programmes that were integrated or disability-specific	Qualitative
Laughlin & Happel, 2016 USA	No participants directly engaged; pertains to students, including blind and partially sighted students, in mainstream education	Goalball	Secondary school PE with an inclusive model	Programme review article
Lepore-Stevens et al., 2021 USA	Blind or partially sighted children and youth and their families	Multi-sport camp	Camp abilities (virtual); disability-specific programming	Practice report
Lieberman et al., 2006 USA	60 blind or partially sighted children and youth; 9–23 years old; recruited from Camp Abilities	Group and individual sports, as well as open and closed	Participants in disability-specific camp sharing experiences from inclusive PE	Qualitative
Lieberman et al., 2009 USA	71 blind or partially sighted children; 38 boys and 33 girls; 9–19 years old; recruited from Camp Abilities	Jump rope	Disability-specific programme	Qualitative
Lieberman, Lepore, Lepore-Stevens, & Ball, 2019 USA	No participants directly engaged; pertinent for physical educators and blind and partially sighted students in mainstream education	Mainstream, adapted, and VI sports	Inclusive PE	Programme review article
Lieberman, Haibach-Beach, Sherwood, & Trad, 2019 USA	10 blind or partially sighted adults; 6 women and 4 men; 23–60 years old	Running	Structured and unstructured participation; road races	Qualitative
Macbeth, 2009 UK	6 blind or partially sighted individuals; White men; 26–40 years old	Football (five-a-side and futsal)	Disability-specific programming at competitive and recreational levels	Qualitative
MacDonald et al., 2020 USA	2 blind or partially sighted athletes and 4 sighted athletes; aged 18–47 years	Goalball	Reverse integration at collegiate level with practice and competition opportunities	Qualitative
Mastro et al., 1986 USA	No participants directly engaged; pertains to blind and partially sighted students in mainstream education	Wrestling	Integrated, competitive and recreational, and school/higher education sport programmes	Programme review article
Mycock & Molnár, 2021 UK	Sighted coach; British	VI/Blind football	Mainstream and disability-specific	Qualitative
Nixon, 1988 USA	Parents of 18 blind or partially sighted children (aged 7–19; 7 male and 11 female); 15 mothers and 9 fathers 14 professionals and volunteers working in the field	Structured and unstructured participation in sport activities	Formally organized sports outside of school at the competitive level	Qualitative
Nixon, 1989 USA	One partially sighted boy; aged 7 when the study began	Sports including track and field and nerf soccer	Competitive and recreational, as well as integrated, programming	Qualitative
Norris et al., 2018 USA	Parents of blind or partially sighted children who participated in programme	Aquatics	Fit Families programme for parents and blind or partially sighted children	Practice report
O’Connell et al., 2006 USA	No participants directly engaged; relates to blind and partially sighted students and physical educators in mainstream education	Mainstream (e.g., swimming) and disability sports (also called blind/VI sports; e.g., goalball)	Inclusive and segregated programmes were mentioned	Programme review article
Ward et al., 2011 USA	8 blind or partially sighted youth; 5 boys and 3 girls; 10–18 years old	Sports including goalball, baseball, and cheerleading	Not specified	Qualitative

**Table 2.** Experiential elements of quality participation.

Citation	Belongingness	Engagement	Autonomy	Mastery	Meaning	Challenge
Barnett et al., 1993	•			•		
Braga et al., 2015		•		•		
Brunes et al., 2017	•	•		•		
Columna et al., 2015	•	•		•	•	
Columna et al., 2017	•	•	•	•	•	
de Schipper et al., 2017	•			•		
Esatbeyoglu et al., 2023	•	•	•	•	•	•
Gombás & Gál, 2016	•	•	•	•		
Goodwin et al., 2011	•	•	•	•	•	•
Green & Miyahara, 2007	•	•	•	•	•	•
Haegele et al., 2014		•	•	•		
Haegele et al., 2017	•	•	•	•	•	•
Hall et al., 2023	•	•	•	•	•	
Kirk & Haegele, 2021	•		•	•	•	
Laughlin & Happel, 2016	•	•	•	•	•	•
Lepore-Stevens et al., 2021	•	•		•	•	•
Lieberman et al., 2006	•		•	•	•	
Lieberman et al., 2009	•	•		•		•
Lieberman, Lepore, Lepore-Stevens, & Ball, 2019	•	•	•	•	•	•
Lieberman, Haibach-Beach, Sherwood, & Trad, 2019		•	•	•		•
Macbeth, 2009	•	•		•		•
MacDonald et al., 2020	•	•		•	•	•
Mastro et al., 1986	•		•	•		•
Mycock & Molnár, 2021	•		•	•	•	•
Nixon, 1988	•			•		•
Nixon, 1989	•			•	•	
Norris et al., 2018			•	•		•
O’Connell et al., 2006			•	•		•
Ward et al., 2011	•	•		•	•	

**Table 3.** Strategies to foster quality participation.

	Belongingness	Engagement	Autonomy	Mastery	Meaning	Challenge
MULTI-SENSORY ENVIRONMENT						
Access to sport equipment – Various multi-sensory equipment options (e.g., bell ball, tactile markings) are available for use in programming and by community members (e.g., equipment lending library, sending equipment to participants). Programme leaders play important roles in ensuring athletes have access to equipment and that creative solutions are identified when equipment is not available/optimal.	Sport equipment that is perceivable and engaging for diverse participants is available, fostering a sense of respect for them as athletes. Having access can address barriers to participation and contribute to feelings of acceptance and belongingness. • de Schipper et al. (2017), Esatbeyoglu et al. (2023), Gombás and Gál (2016), Kirk and Haegele (2021), Lieberman et al. (2009)	The availability of multi-sensory equipment that reflects how blind and partially sighted interact with the world can decrease frustration and help participants stay absorbed in the activity. • Braga et al. (2015), Laughlin and Happel (2016), Lepore-Stevens et al. (2021), Lieberman et al. (2009), Gombás and Gál (2016)	Having a variety of equipment can provide participants with choice. • Gombás and Gál (2016), Kirk and Haegele (2021)		Access to sport equipment ensures that participants can contribute to personally and socially meaningful goals. Collaborative efforts are made with athletes to find creative solutions in the absence of particular equipment. • Columna et al. (2017)	Having access to multi-sensory equipment or working to find creative alternatives supports participants in attaining an appropriate level of challenge, while avoiding frustration due to barriers. • Lepore-Stevens et al. (2021)
Accessibility including environmental modification – At minimum, the facilities are accessible for blind and partially sighted people (e.g., sidewalks leading to facilities, clear walkways in buildings, large print information), programme leaders are prepared to and place importance on making environmental modifications to ensure accessibility. The design and modification of the multi-sensory environment contributes to feelings of safety for participants.	Accessibility and environmental modification ensure all participants are able to participate alongside the group and contribute to the feelings of acceptance. • Kirk and Haegele (2021)			Accessibility and environmental modifications ensure participants’ needs are met, allowing them to improve their skills and feel capable. Examples include larger cones, varying the heights of goals/nets, brighter-coloured goal posts, and adjusted lighting in facility. • Braga et al. (2015), de Schipper et al. (2017), Lieberman et al. (2006), Lieberman et al. (2009)		
Familiar environment – Feeling of safety increases when the facilities are familiar to participants, meaning that they have the opportunity to explore the area, have knowledge of the layout, and know how to navigate.	The environment is safe for participants with diverse abilities and identities, allowing them to participate alongside the group. • Goodwin et al. (2011)	When in a safe and/or familiar environment, participants can be more present in the activity. • Esatbeyoglu et al. (2023), Lieberman, Haibach-Beach, Sherwood, & Trad (2019)	Participants can engage more independently when the environment is safe. Participants are able to navigate and engage with their environment independently when it is familiar and when they feel safe. • Esatbeyoglu et al. (2023), Hall et al. (2023), Lieberman, Haibach-Beach, Sherwood, & Trad (2019)	Programme leaders can ensure that participants have time to acclimatize to the sport environment, allowing participants to achieve their goals without fear. Participants feel more competent and work towards their goals without competing cognitive demands when their environment is familiar. • Barnett et al. (1993), Goodwin et al. (2011), Kirk and Haegele (2021), Lieberman, Haibach-Beach, Sherwood, & Trad (2019), Norris et al. (2018)		Participants can identify and engage with an appropriate level of challenge when in a safe environment. • Esatbeyoglu et al. (2023), Goodwin et al. (2011)
Variation – Various environments are available for programming.	Providing numerous environments during programming contributes to feelings of acceptance and inclusion. • Green and Miyahara (2007), Hall et al. (2023)	Different environments provide a variety of stimulation and feelings that help participants be in the moment. • Hall et al. (2023)	Providing a choice to participants between various environments allows them to be in control. • Hall et al. (2023), Lieberman et al. (2006)			
Environmental feedback and enrichment – Beyond meeting accessibility requirements, multi-sensory elements, such as tactile markers for play boundaries, music, and verbal play-by-play descriptions, can be added to the environment to create rich sport experiences and provide real-time feedback to athletes while participating in sport.	Adding multi-sensory elements that are perceivable and provide ongoing feedback to athletes helps to ensure that blind and partially sighted participants feel they are part of the group and feel that their experiences are valued. • Esatbeyoglu et al. (2023)	Participants are more absorbed in the activity when the environment is interesting and engaging in ways that are perceivable to blind and partially sighted athletes as they navigate through the sport environment and engage in play, increasing feelings of safety and ensuring the activity is engaging for them. Examples include adding musical elements, being outdoors, or using paired movements (tactile). • Braga et al. (2015), Columna et al. (2017), Esatbeyoglu et al. (2023), Hall et al. (2023), Lieberman et al. (2009)				
PROGRAMME						
Representing community – The sport programme allows participants to become representatives of their community (e.g., university).	Participants feel respected as part of the group when they are able to represent their community or group during sport, such as being a recognized university sports team. • MacDonald et al. (2020)				Participants feel they are able to contribute meaningfully as representatives of their communities, particularly by promoting the visibility of disability. • Lieberman, Lepore, Lepore-Stevens, & Ball (2019), MacDonald et al. (2020)	
Preparation and pre-teaching – The preparation of programme leaders is important for quality programming. Programme leaders should provide important information regarding the sport, its rules, and the playing area to athletes before engaging in activities.	When they have a foundational understanding of the activity, participants can follow the programming and participate as part of the group. • Columna et al. (2015), Lieberman et al. (2006)	By engaging in pre-teaching, programme leaders mitigate fatigue and avoid dividing the attention of athletes during activities. Also, this pre-teaching avoids feelings of frustration and being left behind. • Columna et al. (2015), Laughlin and Happel (2016)	Athletes can navigate more independently when they are provided with information about the sport environment using strategies such as tactile maps. Programme leaders can provide opportunities for self-directed exploration of the sport environment. • Mastro et al. (1986), Norris et al. (2018)	Programme leaders can provide navigation and orientation information by using tactile maps prior to the start of programming. Common terminology should be established to ensure effective communication. • Hall et al. (2023), Lieberman et al. (2006), Lieberman, Lepore, Lepore-Stevens, & Ball (2019)	Preparing adequately for activities ensures that all athletes are able to contribute to the programming. Well-developed programming can also integrate meaningful elements, such as the opportunity to gain new knowledge or engage with a new culture. • Columna et al. (2015)	
Multi-sport options – Programmes offer a multi-sport experience making various sport and activity options available to participants.	When provided options, participants feel respected as members of the group and feel that their perspectives are valued. • Columna et al. (2015), Green and Miyahara (2007), Haegele et al. (2017), Lieberman et al. (2009), Mastro et al. (1986), Nixon (1989)	Participants are more absorbed in the moment when they can choose an activity that is most engaging for them. • Haegele et al. (2017), Lepore-Stevens et al. (2021), Lieberman et al. (2006)	Providing various options ensures that participants feel that they have control over their participation. • Haegele et al. (2017), Lieberman et al. (2006), Mastro et al. (1986), Mycock and Molnár (2021)	Being able to choose an activity that is most appropriate for them allows participants to feel more competent. • Braga et al. (2015), Columna et al. (2015), de Schipper et al. (2017), Goodwin et al. (2011), Haegele et al. (2014), Haegele et al. (2017), Kirk and Haegele (2021), Lieberman, Lepore, Lepore-Stevens, & Ball (2019), Lieberman et al. (2006), O’Connell et al. (2006), Nixon (1989), Mycock and Molnár (2021)	When provided options, participants can select the activity that allows them to work towards their goals. • Hall et al. (2023), Green and Miyahara (2007)	Participants can select activities that are most appropriate for them, their needs, and their strengths which allows them to feel adequately challenged without experiencing frustration. • Mastro et al. (1986)
Structure and schedule – The programme provides structure and schedules to participants.	By providing structure and a schedule shared by numerous/all participants, athletes feel part of a group, especially when engaging with virtual programming. • Lepore-Stevens et al. (2021)	Participants feel more in-the-moment when supported by a set schedule and programme structure. • Lepore-Stevens et al. (2021)		Participants are able to feel competent and a sense of achievement when their athletic participation and development is supported by structured sport programming. • Nixon (1988)		
Sport type – The sport type is chosen deliberately, with consideration for the experiences, perspectives, needs, and strengths of participants. Potential risks should also be considered.	The chosen sport should be accessible and facilitate the participation of blind and partially sighted athletes and allow them to be part of the group. Culturally relevant activities are particularly helpful for cultivating acceptance. • Haegele et al. (2017), Hall et al. (2023), Laughlin and Happel (2016), Lieberman et al. (2006), Nixon (1989)	Activities that are most engaging for participants include culturally relevant activities that reflect the experiences of the VI community, novel activities, and activities that allow participants to experience new embodied sensations. Participants are able to focus when they feel the activity is safe. • Esatbeyoglu et al. (2023), Haegele et al. (2017), Lieberman, Lepore, Lepore-Stevens, & Ball (2019)	Sport activities should be chosen with consideration for how blind and partially sighted athletes experience the world to support their ability to participate independently. Particular activities can also provide a freedom of movement that blind and partially sighted athletes do not often have the opportunity to experience in a sighted world. • Esatbeyoglu et al. (2023), Lieberman et al. (2006)	The chosen sport should be appropriate for the strengths and needs of athletes with VI while also mitigating potential risks (e.g., retinal detachment during ball games played at head height). Culturally relevant and/or novel activities are particularly important to cultivate a sense of achievement. • Macbeth (2009), Nixon (1989)	Activities that integrate Paralympic culture, disability-specific sports, or sports that contribute to the visibility of disability are particularly meaningful. • Haegele et al. (2017), Laughlin and Happel (2016), Lieberman et al. (2006), Lieberman, Lepore, Lepore-Stevens, & Ball (2019)	The chosen activity should allow participants to feel challenged as athletes, without being frustratingly difficult and inaccessible. • Lieberman, Lepore, Lepore-Stevens, & Ball (2019), Macbeth (2009)
Accessible policies and rule modifications – The programme accommodates athletes and provides modifications to the rules of activities.	Programmes and their policies should facilitate the participation of blind and partially sighted athletes. The cost to participate in programming is attainable for participants and accommodations for blind and partially sighted athletes should not have additional costs. • Hall et al. (2023)		Athletes should be supported through rule modification and consulted in the modification process. • Lieberman et al. (2006)	Programmes should be accessible to blind and partially sighted athletes and programme leaders should be willing to modify when necessary. Modifications including additional rules (e.g., playing below head height), changes to the flow of play, the addition of tactile elements, and changes to the skills required (e.g., handing off the ball instead of throwing). • Braga et al. (2015), Lieberman et al. (2006), Mastro et al. (1986)		
Individual-level support – Support is provided on an individual level, with consideration for the needs and preferences of each participant.		One-on-one support is helpful to keep athletes absorbed in the activity. • Goodwin et al. (2011)	When one-on-one support is available, athletes are able to have more control over their experiences. • Goodwin et al. (2011)	Athletes develop a sense of achievement when support is provided on an individualized level. • Braga et al. (2015), Brunes et al. (2017), Columna et al. (2017), Haegele et al. (2017), Laughlin and Happel (2016), Lieberman et al. (2006), Lieberman, Lepore, Lepore-Stevens, & Ball (2019), O’Connell et al. (2006), Mastro et al. (1986)	Athletes feel a responsibility to others when working in a pair. • Green and Miyahara (2007), Lieberman, Lepore, Lepore-Stevens, & Ball (2019), Nixon (1989)	Supporters are available to identify an appropriate level of challenge for each athlete, tailoring the activities to each athlete and providing one-on-one support. • Green and Miyahara (2007), Lieberman et al. (2009), Lieberman, Lepore, Lepore-Stevens, & Ball (2019), MacDonald et al. (2020), O’Connell et al. (2006)
SOCIAL – GENERAL						
Multiple supporters – There are numerous different supporters, including coaches, para educators, staff, and guides, available to participants.			Participants have choice in who supports them, how they receive support, and when they are supported. Having multiple guides available also ensures that participants are supported, despite any changes in the availability of supporters. • Green and Miyahara (2007), Lieberman, Haibach-Beach, Sherwood, & Trad (2019)	When multiple supporters are available to provide support, they can be positioned at different locations of the sport environment to support athletes throughout sport participation. • Barnett et al. (1993), de Schipper et al. (2017), O’Connell et al. (2006)		
Group environment – Programme leaders play important roles in fostering a group environment that is accepting, strength-based, and interdependent.	Programme leaders should cultivate feelings on togetherness within the group and a recognition of the strengths of one another. • Barnett et al. (1993), Columna et al. (2017), Esatbeyoglu et al. (2023), Hall et al. (2023), Laughlin and Happel (2016), Macbeth (2009), MacDonald et al. (2020), Mycock and Molnár (2021), Nixon (1989)	It is most engaging when participants experience sensory attunement (i.e., feeling and being aware of the different types of sensations during participation, particularly partnered participation) and there is harmony among the group. • Hall et al. (2023), Macbeth (2009), Lieberman et al. (2006)	Programme leaders can allow participants to have control over the group with whom they are paired. Also, ensuring continuity in the groups can support athletes’ ability to engage independently. • Green and Miyahara (2007), Laughlin and Happel (2016)	Classification of athletes should not be based solely on level of sight or on perceived *deficits*. Athletes should be provided the opportunity to work in pairs and develop relational efficacy. • Barnett et al. (1993), Hall et al. (2023), Haegele et al. (2014), Laughlin and Happel (2016), MacDonald et al. (2020), Macbeth (2009), Nixon (1989)	All members of the group feel a responsibility to be mutually supportive, cultivate the accessibility and safety of the activity, and ensure all athletes can independently engage. The group environment emphasizes participation over competition and winning. • Laughlin and Happel (2016), Nixon (1989), Mycock and Molnár (2021)	
Status of disability – The disability-related attitudes of programme leaders influence the status of disability within the sport context, creating an environment of equity or ableism (or a combination of both).	Programme leaders should reject assumptions about how blindness and partially sight impacts the experiences of athlete and their needs in sport context. Disability should not be blamed for an athletes’ skill level or mistakes. Blind and partially sighted athletes feel more accepted as part of the group when disability is defined more interpersonally and experientially, rather than focused on the medical and rehabilitation models. • Green and Miyahara (2007), Goodwin et al. (2011), Kirk and Haegele (2021), Lieberman, Lepore, Lepore-Stevens, & Ball (2019), Mastro et al. (1986), Nixon (1989)	The attitudes towards disability should foster a safe social environment, free from ableism, to allow participants to stay more focused on the activity, as opposed to worrying about discrimination and the need to defend themselves. • Esatbeyoglu et al. (2023), Goodwin et al. (2011), Lieberman, Lepore, Lepore-Stevens, & Ball (2019)	Programme leaders should affirm an athlete’s experiences of disability as valid and recognize the diverse ways of engaging with the world that do not rely solely on sight, as opposed to viewing blindness and partial sight as a *deficit*. • Goodwin et al. (2011), Green and Miyahara (2007)	Programme leaders should not blame low performance on an athlete’s disability and should not see their disability as determining the among they can achieve. • Green and Miyahara (2007), Goodwin et al. (2011), Nixon (1989), O’Connell et al. (2006)	The enjoyment and participation of all athletes should be valued, and all people should feel a responsibility to support the participation of one another. • Columna et al. (2015), Goodwin et al. (2011), Green and Miyahara (2007), Kirk and Haegele (2021)	Programme leaders should expect an appropriate level from athletes based on their individual abilities, rather than based on assumptions. Athletes should be challenged, not overprotected. • Haegele et al. (2017), Goodwin et al. (2011), Lieberman, Lepore, Lepore-Stevens, & Ball (2019), MacDonald et al. (2020), Nixon (1988), O’Connell et al. (2006)
Diversity – The social environment welcomes and recognizes that every athlete is different. The participation of diverse athletes should be supported.	Programme leaders should create an environment that values differences and uniqueness, minimizing the othering experienced by blind and partially sighted athletes and other marginalized identities. • Green and Miyahara (2007), Lieberman, Lepore, Lepore-Stevens, & Ball (2019), MacDonald et al. (2020), Nixon (1989)			Programme leaders should create opportunities for athletes to feel comfortable and be able to communicate their needs, particularly in cases where such needs are not visible, to ensure they receive the support they need to feel competent. • Columna et al. (2017), Goodwin et al. (2011), Lieberman, Lepore, Lepore-Stevens, & Ball (2019), MacDonald et al. (2020), Mycock and Molnár (2021), Nixon (1989)	Diversity is particularly meaningful when differences are embraced, recognizing that each athlete contributes uniquely to the sport programme and are deserving of support. • Columna et al. (2015), Columna et al. (2017), Goodwin et al. (2011), Green and Miyahara (2007), Haegele et al. (2017), Kirk and Haegele (2021) Lieberman, Lepore, Lepore-Stevens, & Ball (2019), MacDonald et al. (2020), Nixon (1989)	
Encouragement – Encouragement is an important part of the sport experience, provided by programme leaders, supporters, and peers.	Providing encouragement to one another contributes to feelings of acceptance. • Ward et al. (2011)	Verbal encouragement provided to athletes helps to keep them focused on the activity and enriches the multi-sensory environment. • Columna et al. (2017)		Providing encouragement to athletes contributes to their feelings of competence and supplements other task-specific feedback to support skill development. • Columna et al. (2017), Esatbeyoglu et al. (2023), Kirk and Haegele (2021), Nixon (1989), Ward et al. (2011)	Programme leaders should encourage athletes in ways that are genuine, as opposed to attitudes that may come from low expectations, ableism, or objectification. • Kirk and Haegele (2021)	
Forming reciprocal relationships – Programme leaders should encourage and support participants, staff, peers, and volunteers to form relationships that are mutually supportive and equal.	Participants experience a sense of belongingness when they are able to contribute to the relationships they build, receiving and giving support. Participants also value the ability to reconnect with the individuals they form relationships with at future programming. • Esatbeyoglu et al. (2023), Goodwin et al. (2011), Hall et al. (2023), Kirk and Haegele (2021), Lepore-Stevens et al. (2021), MacDonald et al. (2020), Mycock and Molnár (2021)	Relationships that foster sensory attunement between partners are most engaging, although safety should remain a focus even when partners experience flow during activities. • Esatbeyoglu et al. (2023), Goodwin et al. (2011), Hall et al. (2023), Lepore-Stevens et al. (2021)		Participants feel more competent when they develop relational efficacy with a partner through co-active movement and sensory attunement. • Goodwin et al. (2011), Green and Miyahara (2007), Haegele et al. (2014), Hall et al. (2023), MacDonald et al. (2020), Nixon (1989), Mycock and Molnár (2021)	Creating opportunities for athletes to form relationships is meaningful, particularly for athletes who are blind or partially sighted who can experience social isolation. Also, athletes feel a sense of responsibility to others when they are able to form relationships. • Goodwin et al. (2011), Hall et al. (2023), Mycock and Molnár (2021), Nixon (1989), Ward et al. (2011)	
Unique pathways – Participants are able to develop their own path through sport, across different activities and levels.			Participants should have control over their pathway as athletes including level of competition, and sport type. • Green and Miyahara (2007), Haegele et al. (2017), Mastro et al. (1986)	The individual goals of participants should be considered, and opportunities for progression should be offered. • Lieberman et al. (2009), Macbeth (2009), Mastro et al. (1986), Nixon (1989)	Programme leaders should have knowledge of the paralympic sport programmes available for athletes wishing to become more involved in the paralympic community. • Lieberman et al. (2006)	Athletes should be encouraged to participate at levels that suit their abilities and goals, progressing to an appropriate level of challenge. • Macbeth (2009), Mastro et al. (1986)
SOCIAL – PROGRAMME LEADER						
Multi-sensory instruction and feedback – To ensure the growth of the athlete, instructors should provide instruction using various multi-sensory techniques and give ongoing feedback in ways that are helpful for the athlete.	Programme leaders can affirm athletes as valued members of the group when they receive instruction and feedback that is appropriate. • Barnett et al. (1993), Esatbeyoglu et al. (2023), Hall et al. (2023), Lieberman et al. (2006), Mastro et al. (1986)	Programme leaders engage athletes when they provide instruction and feedback that is appropriate. • Columna et al. (2017), Goodwin et al. (2011), Lieberman, Lepore, Lepore-Stevens, & Ball (2019), MacDonald et al. (2020)	Programme leaders can provide athletes with various choices for instruction. Also, programme leaders and athletes can develop unique strategies together to fit their needs and the sport context. • Haegele et al. (2014), Lieberman, Lepore, Lepore-Stevens, & Ball (2019), O’Connell et al. (2006)	Programme leaders should provide task-specific feedback to support an athlete’s development and sense of achievement. This can be verbal, tactile, or any other communication strategy developed with the athlete. • de Schipper et al. (2017), Esatbeyoglu et al. (2023), Goodwin et al. (2011), Haegele et al. (2014), Hall et al. (2023), Laughlin and Happel (2016), Lieberman, Lepore, Lepore-Stevens, & Ball (2019), Lieberman et al. (2006), O’Connell et al. (2006), Mastro et al. (1986), Mycock and Molnár (2021)		
Autonomy support – Programme leaders should work collaboratively with athletes to ensure their voices are heard and their needs, feelings, and preferences are recognized to co-create a positive sport experience.		Programme leaders should tailor instruction to the participant by eliciting their goals as athletes. • Columna et al. (2017), Goodwin et al. (2011), Lieberman et al. (2006)	Programme leaders should provide athletes with options and, when using tactile and physical instructional techniques, should promote bodily autonomy. • Columna et al. (2017), Goodwin et al. (2011), Green and Miyahara (2007), Haegele et al. (2017), Hall et al. (2023), Lieberman et al. (2006), Lieberman, Lepore, Lepore-Stevens, & Ball (2019), Mycock and Molnár (2021), O’Connell et al. (2006)	Provide the athlete with numerous options for instructional techniques. A person-centred approach to coaching is particularly useful. • de Schipper et al. (2017), Haegele et al. (2014), Laughlin and Happel (2016), Lieberman et al. (2006), O’Connell et al. (2006), Mycock and Molnár (2021), Mastro et al. (1986), Nixon (1989)		Programme leaders should be supportive without being overprotective or intrusive, allowing athletes to control over the level of challenge that is most appropriate for them. • Haegele et al. (2017), Goodwin et al. (2011), MacDonald et al. (2020), O’Connell et al. (2006)
Role development – Instructors should create roles and recognize the roles created by athletes, allowing athletes the experience each role.	Participants should be provided the opportunity to contribute to the group in various roles including in demonstrations, as referees, as planners, and as leaders. • Green and Miyahara (2007), Lieberman et al. (2006), Lieberman et al. (2009), Lieberman, Lepore, Lepore-Stevens, & Ball (2019), MacDonald et al. (2020), Mastro et al. (1986)	Programme leaders should develop roles that are of interest to athletes and recognize the roles defined by the athletes. • Green and Miyahara (2007), Laughlin and Happel (2016), MacDonald et al. (2020)		Programme leaders should recognize the contributions of athletes while in each role. • Laughlin and Happel (2016), Lieberman, Lepore, Lepore-Stevens, & Ball (2019), MacDonald et al. (2020), Mastro et al. (1986)	Athletes feel they can contribute to meaningful goals when in each role. • Green and Miyahara (2007), Hall et al. (2023), MacDonald et al. (2020)	
Interpersonal skills – Programme leaders should foster positive relationships with participants and bring excellent interpersonal skills as a leader.	Programme leaders should promote a positive and trusting group environment that is accepting of all athletes. • Columna et al. (2015), Esatbeyoglu et al. (2023), Goodwin et al. (2011)		Programme leaders should cultivate a space in which athletes feel comfortable sharing their needs and provide options to participants. • Columna et al. (2017), Goodwin et al. (2011), Green and Miyahara (2007), Haegele et al. (2017), Hall et al. (2023), Lieberman et al. (2006), Lieberman, Lepore, Lepore-Stevens, & Ball (2019), Mycock and Molnár (2021), O’Connell et al. (2006)	Programme leaders should engage in open communication about the preferences and needs of athletes to support their development. • Columna et al. (2017), Esatbeyoglu et al. (2023), Hall et al. (2023), Laughlin and Happel (2016), Lieberman et al. (2006), MacDonald et al. (2020), Mastro et al. (1986)	Programme leaders should demonstrate genuineness and patience when engaging with athletes. • Esatbeyoglu et al. (2023), Goodwin et al. (2011)	Programme leaders should cultivate an environment in which athletes are comfortable providing input into the level of challenge they experience. • Haegele et al. (2017), MacDonald et al. (2020)
Knowledge, skills, and learning – Instructors should have adequate knowledge, skills, and learning to design appropriate programming and support diverse athletes.	Programme leaders are integral in promoting feelings of acceptance and inclusion within the group. • Brunes et al. (2017), Gombás and Gál (2016), Haegele et al. (2017), Kirk and Haegele (2021), Laughlin and Happel (2016), Lieberman et al. (2006)	Programme leaders should consider how athletes experience the activity to provide optimally engaging programming, integrating the use of technology when applicable. By providing practical and navigation support, programme leaders allow athletes to focus solely on the activity. • Brunes et al. (2017), Columna et al. (2017), Esatbeyoglu et al. (2023), Gombás and Gál (2016), Goodwin et al. (2011), Haegele et al. (2014), Haegele et al. (2017), Laughlin and Happel (2016), Lepore-Stevens et al. (2021), Lieberman, Lepore, Lepore-Stevens, & Ball (2019)	When programme leaders have adequate knowledge and skills, they are able to offer numerous instructional techniques for athletes to choose form. Additionally, athletes should be provided control over their participation and have autonomy to define themselves as athletes. • Columna et al. (2017), Green and Miyahara (2007), Goodwin et al. (2011), Haegele et al. (2014), Haegele et al. (2017), Lieberman et al. (2006), Lieberman, Lepore, Lepore-Stevens, & Ball (2019)	Programme leaders use effective instructional methods, such as whole part whole, repetitive practice, and physical guidance, to support athletes in developing a sense of competence. • Columna et al. (2017), de Schipper et al. (2017), Esatbeyoglu et al. (2023), Haegele et al. (2014), Hall et al. (2023), Lieberman et al. (2006), Lieberman et al. (2009), MacDonald et al. (2020), Mastro et al. (1986), O’Connell et al. (2006)		Programme leaders should provide participants with an appropriate level of challenge based on progression of skill and varying degrees of complexity for tasks. • Goodwin et al. (2011), Laughlin and Happel (2016), Lieberman et al. (2009), Lieberman, Lepore, Lepore-Stevens, & Ball (2019), MacDonald et al. (2020), Mycock and Molnár (2021), Nixon (1988)
Exploratory and collaborative learning – The athlete and the programme leader recognize their roles as both knower and learner to co-create a positive sport experience.	Programme leaders should elicit the opinions of participants and integrate them into programming on an ongoing basis, especially since athletes may not have previous experiences with sport. • Goodwin et al. (2011), Mycock and Molnár (2021)	Open communication between programme leaders and participants is essential, allowing athletes to share their expertise and educate others. • Columna et al. (2017), Goodwin et al. (2011)	By approaching instruction in this manner, the athlete has control over their learning. • Esatbeyoglu et al. (2023), Goodwin et al. (2011), Lieberman, Lepore, Lepore-Stevens, & Ball (2019), Lieberman et al. (2006), O’Connell et al. (2006), Mycock and Molnár (2021), Norris et al. (2018)	Programme leaders should support participants in exploring their abilities and potential through guided discovery, which helps programme leaders learn more about how to effectively support the athlete. • Lieberman et al. (2006), Nixon (1989)	The reciprocity of this relationship helps participants feel a sense of responsibility to others. • Goodwin et al. (2011)	Through exploration, athletes can identity an appropriate level of challenge and programme leaders can form appropriate expectations for the athlete’s abilities. Working together, they can continually re-evaluate the level of challenge as they progress through programming. • Goodwin et al. (2011), Lepore-Stevens et al. (2021), Mastro et al. (1986)
Tracking progress – The athlete’s progress, including changes to the level of support, skill development, and speed, should be noted and highlighted. These goals should be defined by the athlete, not based on normativity.		Programme leaders should record progress and communicate specific feedback to athletes to maintain their interest in the programming. • Goodwin et al. (2011)		Communicating the athlete’s progress cultivates a sense of achievement and competence. • Goodwin et al. (2011), Haegele et al. (2014), Lieberman et al. (2006), Lieberman et al. (2009), Nixon (1989), Norris et al. (2018), O’Connell et al. (2006)	Highlighting an athlete’s progress affirms their progress towards meaningful goals. Also, providing specific milestone in an athlete’s progress may introduce insincerity to the feedback. • Goodwin et al. (2011), Nixon (1989)	Tracking and communicating an athlete’s progress serves as an important exercise to evaluate whether the level of challenge is appropriate and if progressions may be helpful. • Laughlin and Happel (2016), Lieberman et al. (2009), Norris et al. (2018), O’Connell et al. (2006)
SOCIAL – GUIDES						
Knowledge, skills, learning (Guides) – Guides should be knowledgeable and prepared to support athletes, with an understanding of their role.		Guides should be trained to effectively support athletes to contribute to a safe environment in which athletes can remain in the moment while participating. • Lieberman, Haibach-Beach, Sherwood, & Trad (2019)				
SOCIAL – FAMILY						
Family integration – Parents and siblings are important to creating an accepting group environment, when programme leaders ensure that athletes remain independent.	Programme leaders can foster a sense of acceptance among the group when families are invited to become involved in programming. • Columna et al. (2017), Lepore-Stevens et al. (2021), Nixon (1988), Nixon (1989)	Programme leaders can involve parents and siblings in engaging athletes during sport. Culminating events at the end of programmes are particularly useful. • Laughlin and Happel (2016), Lepore-Stevens et al. (2021)		Families can contribute to feelings of competence, particularly during virtual programmes. • Lepore-Stevens et al. (2021)	Including siblings and parents in programming can be meaningful for participants. • Lepore-Stevens et al. (2021)	
SOCIAL – DOG						
Support from guide dogs – Programme leaders can create the opportunity for athletes to include their guide dogs in programming.	Guide dogs can encourage social interactions with others in sport contexts. • Green and Miyahara (2007)	Guide dogs provide navigation and orientation support to athletes to allow them to focus on skill development and mitigate fatigue during participation. • Green and Miyahara (2007), Lieberman, Haibach-Beach, Sherwood, & Trad (2019)	Guide dogs can support the independence of athletes by providing navigation and orientation support. Programme leaders can offer numerous options related to when and where athletes involve guide dogs. • Lieberman, Haibach-Beach, Sherwood, & Trad (2019)	Athletes can develop a sense of achievement, supported by their guide dog during sport programming. • Lieberman, Haibach-Beach, Sherwood, & Trad (2019)		Guide dogs can provide the one-on-one support required for athletes to find an appropriate level of challenge. • Lieberman, Haibach-Beach, Sherwood, & Trad (2019)
SOCIAL – PEERS						
Group (blind and partially sighted peers) environment – In disability-specific programming, participants are able to build shared culture, feel understood, and feel safe.	Participating alongside blind and partially sighted peers (i.e., that share their experiences) contributes to feelings of acceptance and being a member of a group. • Goodwin et al. (2011), Green and Miyahara (2007), Haegele et al. (2017), Kirk and Haegele (2021), Lepore-Stevens et al. (2021)			Programme leaders should encourage peers to support one another to achieve their goals and develop as athletes. • de Schipper et al. (2017)	Participants should be encouraged to develop shared goals during sport programming. • Green and Miyahara (2007), Haegele et al. (2017), Goodwin et al. (2011), Lieberman et al. (2006)	
Representation, mentorship, and role modelling – Opportunities to receive mentorship, provide mentorship, and be role models are provided to athletes.	Participants feel they belong when they see themselves represented within the sport environment, when they are able to be role models for other athletes with VI, and when sighted peers value their presence as authentic representation of the VI community. • Goodwin et al. (2011)		Representation and role models help to empower athletes to exercise more independence. • Goodwin et al. (2011)	Participating alongside other blind and partially sighted athletes empowers participants to feel competent and achieve their goals. • Goodwin et al. (2011)	Having the opportunity to mentor others or be authentic representation of blind and partially sighted athletes and for other blind and partially sighted athletes. • Goodwin et al. (2011), Lepore-Stevens et al. (2021)	
Group (sighted peer) environment – Programme leaders should cultivate an environment that values one another, respects differences, and builds a mutual awareness of the diverse experiences of participants.	To cultivate feelings of acceptance, programme leaders should encourage an environment of learning and willingness to learn about the experiences of blind and partially sighted athletes. Sighted peers contribute to feelings of acceptance by providing support, such as verbal descriptions of activities and navigation information, to blind and partially sighted peers. • Kirk and Haegele (2021), Laughlin and Happel (2016), Lieberman et al. (2006), Lieberman, Lepore, Lepore-Stevens, & Ball (2019), Mastro et al. (1986)	The willingness of sighted peers to learn about the experiences of blind and partially sighted athletes and the ability of blind and partially sighted athletes share about their experience encourages athletes to be in the moment. • Laughlin and Happel (2016)	Participants should be encouraged to support one another to create opportunities for peers to be independent. • Laughlin and Happel (2016)		Programme leaders should encourage peers to define shared goals and cultivate interdependence with one another. Peers should also be encouraged to learn from one another and demonstrate a willingness to learn about blindness and partial sight. • Goodwin et al. (2011), Hall et al. (2023)	
SOCIAL – REFEREES AND OFFICIALS						
Multi-sensory feedback/calls – As integral parts of sport contexts, referees and officials should also be encouraged to facilitate the participation of blind and partially sighted athletes.				Referees should recognize the importance of communicating in ways that are understandable for participants. • Mastro et al. (1986)		

**Table 4.** Outcomes associated with quality participation.

Outcomes	Belongingness	Engagement	Autonomy	Mastery	Meaning	Challenge	None
EXPERIENTIAL							
Enjoyment and fun	Columna et al. (2015), Columna et al. (2017), Kirk and Haegele (2021), MacDonald et al. (2020)	Columna et al. (2017), Esatbeyoglu et al. (2023)	Green and Miyahara (2007)	Brunes et al. (2017), Columna et al. (2015)	Green and Miyahara (2007), MacDonald et al. (2020)		Brunes et al. (2017), Lepore-Stevens et al. (2021), Mycock and Molnár (2021), Nixon (1988), Norris et al. (2018)
Enthusiasm for sport participation	Columna et al. (2017), Kirk and Haegele (2021), Lepore-Stevens et al. (2021)	Braga et al. (2015), Ward et al. (2011)		Braga et al. (2015), Laughlin and Happel (2016)	Green and Miyahara (2007)		
Mental and physical benefits	Green and Miyahara (2007)	Braga et al. (2015), Green and Miyahara (2007), Ward et al. (2011)	Lieberman, Haibach-Beach, Sherwood, & Trad (2019)	Braga et al. (2015)			Columna et al. (2015), Columna et al. (2017), Hall et al. (2023), Kirk and Haegele (2021), Lieberman et al. (2009), MacDonald et al. (2020), Mycock and Molnár (2021), Nixon (1989)
Increased participation in sport/PA and adoption of other healthy habits		Columna et al. (2017)			Green and Miyahara (2007)		Gombás and Gál (2016), Haegele et al. (2017), Laughlin and Happel (2016), Lieberman et al. (2009)
LEARNING AND SKILL DEVELOPMENT							
Knowledge (of sport, self, and accommodations)	Esatbeyoglu et al. (2023), Green and Miyahara (2007)	Braga et al. (2015), Goodwin et al. (2011), Green and Miyahara (2007)	Goodwin et al. (2011)	Braga et al. (2015), Esatbeyoglu et al. (2023), Goodwin et al. (2011), Kirk and Haegele (2021), Lieberman et al. (2006)	Esatbeyoglu et al. (2023), Goodwin et al. (2011), Green and Miyahara (2007)	Goodwin et al. (2011)	Haegele et al. (2017), Laughlin and Happel (2016), Lepore-Stevens et al. (2021)
Personal skill development and growth		Braga et al. (2015)		Braga et al. (2015)	Green and Miyahara (2007)		Columna et al. (2017), Gombás and Gál (2016), Lepore-Stevens et al. (2021), Lieberman, Haibach-Beach, Sherwood, & Trad (2019), Macbeth (2009), Nixon (1988)
Motor skills, mobility, and balance	Esatbeyoglu et al. (2023)	Esatbeyoglu et al. (2023)		Esatbeyoglu et al. (2023), O’Connell et al. (2006)			Norris et al. (2018), Mastro et al. (1986)
Advocacy skills				Kirk and Haegele (2021)			Haegele et al. (2017), Gombás and Gál (2016)
PERCEPTION OF SELF							
Self-esteem, self-efficacy, and pride	Columna et al. (2017), Green and Miyahara (2007), Hall et al. (2023), Kirk and Haegele (2021)	Goodwin et al. (2011), Green and Miyahara (2007)	Columna et al. (2015)	Gombás and Gál (2016), Goodwin et al. (2011), Kirk and Haegele (2021), Lieberman et al. (2006), O’Connell et al. (2006), Ward et al. (2011)	Goodwin et al. (2011), Green and Miyahara (2007)		Haegele et al. (2017), MacDonald et al. (2020), Mastro et al. (1986), Mycock and Molnár (2021), Nixon (1989)
Connection to body and embodied awareness	Kirk and Haegele (2021)	Esatbeyoglu et al. (2023)	Esatbeyoglu et al. (2023)	O’Connell et al. (2006)	Esatbeyoglu et al. (2023)		
Independence, self-determination, and empowerment	Goodwin et al. (2011), Green and Miyahara (2007)	Green and Miyahara (2007)	Haegele et al. (2014), Goodwin et al. (2011), Lieberman, Haibach-Beach, Sherwood, & Trad (2019), Mycock and Molnár (2021)	Haegele et al. (2014), Goodwin et al. (2011), Laughlin and Happel (2016), Lieberman et al. (2006)	Goodwin et al. (2011)	Goodwin et al. (2011)	Columna et al. (2015), Columna et al. (2017), Lieberman et al. (2009), Nixon (1988)
Athletic expression and identity		Goodwin et al. (2011), Haegele et al. (2017)	Haegele et al. (2017)	Goodwin et al. (2011), Haegele et al. (2017)		Goodwin et al. (2011), Haegele et al. (2017)	Haegele et al. (2017), Lepore-Stevens et al. (2021), Lieberman et al. (2009), MacDonald et al. (2020), Nixon (1988), Ward et al. (2011)
CONNECTIONS WITH OTHERS							
Social connectedness, acceptance, and friendship	Columna et al. (2017), de Schipper et al. (2017), Esatbeyoglu et al. (2023), Goodwin et al. (2011), Green and Miyahara (2007), Haegele et al. (2017), Hall et al. (2023), Kirk and Haegele (2021), Lieberman et al. (2006), MacDonald et al. (2020), Ward et al. (2011)	Esatbeyoglu et al. (2023), Green and Miyahara (2007), Hall et al. (2023), Lieberman, Lepore, Lepore-Stevens, & Ball (2019)	Goodwin et al. (2011), Lieberman, Haibach-Beach, Sherwood, & Trad (2019)	de Schipper et al. (2017), Esatbeyoglu et al. (2023), Hall et al. (2023), Lieberman, Lepore, Lepore-Stevens, & Ball (2019)	Esatbeyoglu et al. (2023), Goodwin et al. (2011), Green and Miyahara (2007), Hall et al. (2023), MacDonald et al. (2020)		Columna et al. (2015), Gombás and Gál (2016), Haegele et al. (2017), Lepore-Stevens et al. (2021), Lieberman et al. (2009), Nixon (1988), Nixon (1989)
Team building, group cohesion, and shared confidence	MacDonald et al. (2020)				MacDonald et al. (2020)		
Connection to blind/partial sight community	Goodwin et al. (2011), Green and Miyahara (2007)			Goodwin et al. (2011)	Goodwin et al. (2011), Green and Miyahara (2007)		Haegele et al. (2017), Kirk and Haegele (2021), Lepore-Stevens et al. (2021)
Connection to family and positive familial relationships	Columna et al. (2015), Columna et al. (2017)						

### Data synthesis and analyses

The first author, who is a queer disabled/neurodivergent master’s student of Irish descent, led the interpretive process of data analysis, in collaboration with the sixth author, who acted as a critical friend (Coghlan & Brydon-Miller, 2014) and mentor. Engaging with the 29 included studies, we conducted our analysis, which were mainly deductive. For each study, we began by extracting the support strategies described within each study, as well as the experiences these strategies foster during active participation. These experiences were mapped onto the QP framework based on direct references to the experiential elements of QP and indirect references to the conceptualization of each element. While this did not occur, any experiential elements not currently represented within the QP framework but nonetheless fostered by support strategies would have been highlighted. We then combined these tables of strategies into six larger tables, one for each of the experiential elements of QP, and consolidated them to remove any repetition. Informed by the QP framework (Evans et al., 2018), we then grouped the strategies within each table based on the foundation strategies currently represented within the QP framework and compiled the remaining strategies (i.e., those not reflective of the foundational strategies) to ultimately generate the 33 total strategies found in Table 3. We then added headings within the tables to further organize the strategies according to the environment to which they pertained (e.g., multi-sensory, programme, social).

Moreover, we conducted inductive analysis using reflexive thematic analysis (Braun & Clarke, 2019, 2021). We engaged with Braun and Clarke’s (2006) six stages of thematic analysis, supported by ongoing reflexivity on the part of the researcher, to ultimately construct three themes that emphasize the broader material and immaterial considerations related to the QP of blind and partially sighted athletes during sport. To do so, we considered the ways that authors in the literature positioned and defined effective support strategies, how they conceptualized the sport participation of blind and partially sighted people, and the points of convergence and divergence between the QP framework in its current form and these definitions and conceptualizations. We suggest that these themes can be adopted by PLs as guiding principles that, together, represent an ethos based on a reconceptualization of sport participation that may help to affirm the experiences and identities of blind and partially sighted athletes.

## Results

### Study characteristics

Of the 29 total studies published between 1986 and 2023, 18 were qualitative, five were programme review articles, three were practice reports, two were mixed methods, and one was quantitative (see Table 1). One study (Hall et al., 2023) directly cited the QP framework, relating their findings to the conception of belongingness in parasport contexts. Across all studies, 378 blind or partially sighted people directly participated in research, although numerous authors highlighted that other blind or partially sighted people were engaged throughout their respective research projects (e.g., advisory capacities, provided feedback). Using the age groups defined by the authors, nine studies reported on the experiences of adults (ages 18–67), although only one specifically investigated the experiences of older adults (ages 53–70), and eight studies pertained to the experiences of children and youth (ages 7–23). Of the 23 studies that recruited and engaged participants, four studies had no mention of sex or gender, while six studies directly reported either sex or gender. The other 13 studies alluded to the gender of some or all of the participants through the reporting of gendered pronouns (e.g., he/him) or gendered labels (e.g., mother, boy). Moreover, only six of the 23 studies discussed the race or ethnicity of participants, with an additional two studies reporting solely nationality. Three studies presented the perspective of sighted athletes and the perspectives of blind and partially athletes, and one study specifically explored the experiences of a sighted coach. Although parent-centred studies were excluded, three included articles presented the perspectives of parents on the sport experiences and participation of their blind or partially sighted children and youth. Seven articles discussed various residential sport camps for blind or partially sighted people – six pertaining to the Camp Abilities programme and the remaining study mentioning the Sport Education Camp. From the six studies mentioning Camp Abilities, three focused on the experiences of campers and/or the programming model employed at camp, while the other three recruited participants from attendees of the camp.

### Experiential elements of QP

Across each of the included articles, all six of the QP experiential elements outlined in the QP framework (Evans et al., 2018) were referenced, either directly or indirectly. Direct mentions included the use of exact terminology (e.g., autonomy, mastery) or references to QP-related literature, while indirect references implicated the definitions of the experiential elements and associated strategies, as outlined in the QP framework (Evans et al., 2018). During data analysis, we synthesized no additional experiential elements. More specifically, we categorized 24 studies as relating to the experiential element of belongingness, with one study (Hall et al., 2023) directly referencing the QP conceptualization of belongingness provided by Evans and colleagues (2018). Mainly without citing Evans and colleagues (2018), we interpreted the following numbers of studies as relating to the remaining experiential elements: 19 studies related to engagement; 17 studies related to autonomy; 29 studies related to mastery; 16 studies related to meaning; and 16 studies related to challenge.

### Principles and strategies for fostering QP

**Figure 2.**
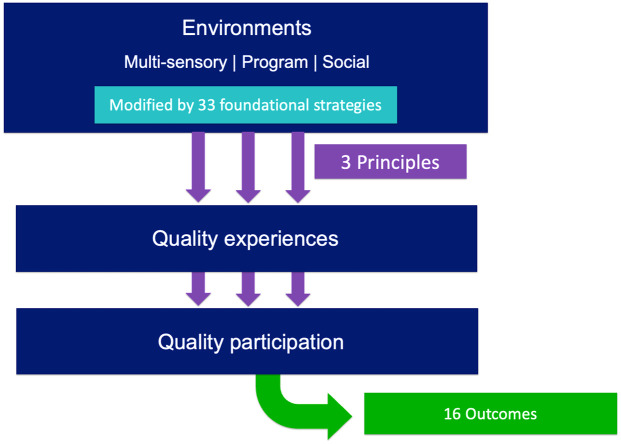
Visual depiction of generated principles within the quality parasport participation framework.

During data analysis, we generated the following three themes that are positioned within this review as principles that may be able to guide PLs during the development and provision of quality sport programming for blind and partially sighted people (see Figure 2 for a visual depiction): (a) consider the multi-sensory experiences associated with sport participation; (b) embrace and respect differences; and (c) embed personalization into programming. These three principles form an ethos that PLs may be able to use to inform their engagement throughout each stage of the QP framework (e.g., development and provision of quality sport programming). More specifically, we offer 33 strategies (see Table 3) based on our engagement with the literature, which we suggest represent specific examples of how to conceptualize and integrate these principles. By adopting this ethos, PLs may be able to reinforce a disability-centred conception of sport participation and affirm the experiences of blind and partially sighted athletes.

The first principle, *consider the multi-sensory experiences associated with sport participation*, reflects the numerous ways in which athletes experience sport. Although these dimensions are often overlooked in favour of the visual elements of participation, various sensations (e.g., embodied, auditory, tactile) are important aspects of sport experiences for many athletes and may be particularly important to conceptualize the experiences of blind and partially sighted athletes in sport contexts. The second principle is *embrace and respect differences*. By doing so, PLs may be able to cultivate an environment of safety and acceptance, encouraging participants to communicate their needs and share their unique perspectives. The last principle, *embed personalization into programming*, describes the uniqueness of each athlete, their diverse needs, and their individual preferences. This final principle encourages PLs to consider the individuality and diversity of the athletes they will serve throughout the creation, planning, and provision of programming to cultivate a culture and programme that is conducive to individualized support and accommodation. As such, PLs must consider these individual-level factors to cultivate a supportive and quality experience for all athletes while minimizing the othering experienced blind and partially sighted athletes. Together, these key principles describe a foundational perspective to guide one’s engagement with and implementation of the strategies presented (see Table 3).

Generated based on the strategies and experiences discussed within the 29 included studies, we outline 33 strategies that foster QP for blind and partially sighted athletes. Many of the 33 strategies foster multiple experiential elements, although 26 were specifically linked with belongingness, 23 with engagement, 21 with autonomy, 27 with mastery, 20 with meaning, and 13 with challenge. Each experiential element was associated with at least one strategy within each of the environments that comprise sport programming: multi-sensory (i.e., facilities and features of the environment; referred to in previous blueprints as physical), programme (i.e., programming-specific considerations), and social (i.e., aspects of the relationships and group dynamics) environments.

### Outcomes

By engaging with the three principles and the associated strategies presented above, PLs may be able to foster QP for blind and partially sighted athletes, culminating in various positive outcomes. Of the 29 studies included in this review, 27 studies were categorized as discussing outcomes of QP for blind and partially sighted athletes. Table 4 displays the 16 outcomes organized in the following four groups: experiential, learning and skill development, perceptions of self, and connections with others. These outcomes represent both acute experiences associated with QP, as well as benefits related to longer-term engagement in quality sport experiences. However, the studies classified these acute and long-term outcomes inconsistently. For example, Hall and colleagues (2023) described the physical benefits resulting from quality sport experiences as a precursor for increased sport participation, while Kirk and Haegele (2021) emphasized the opposite relationship.

## Discussion

This review explored the available literature pertaining to the sport experiences of blind and partially sighted people, as well as the support strategies associated with quality experiences and the outcomes of such participation. By critically engaging with the 29 included studies, we were able to determine that the QP framework, as outlined by Evans and colleagues (2018), was an appropriate framework through which to analyse the collected literature, with direct and indirect mentions to the experiential elements within each study. From the 29 studies included in this review, we generated three principles to support PLs in the provision of quality sport programmes. These principles represent novel contributions to the literature and extend the current QP framework (Evans et al., 2018) to reflect the experiences, perspectives, and support needs of blind and partially sighted athletes.

The first principle that PLs can engage to foster the six experiential elements of QP for blind and partially sighted athletes is to recognize the multi-sensory experiences associated with sport participation. This principle reflects the importance of reconstructing dominant understandings of sport participation to consider the embodied, affective, and aesthetic aspects of participation (Hall et al., 2023; Lynch et al., 2021; Powis & Macbeth, 2024). Some scholars have suggested that, to reconstruct these dominant understandings, it may be pertinent to reject achievement-centred goals that reinforce ableism and elitism and instead consider the embodied- and sensation-related features of participation when determining goals and achievement milestones (Lynch et al., 2021; Powis & Macbeth, 2024). This is particularly important given the ableist roots of mainstream sport (Spencer-Cavaliere et al., 2017) and the hegemonic understandings of sport participation based on visual aspects that are unreflective of the experiences and perspective of blind and partially sighted people (Oldörp et al., 2024; Powis, 2020). Congruent with the aspect of an ableism-critical approach (Oldörp et al., 2024), PLs may be able to engage this principle as they reject implicit ableist constructions of the body and instead consider the unique ways that blind and partially sighted people subjectively experience sport and QP.

To further reconstruct dominant understandings of sport participation, the second principle, *embrace and respect differences*, represents the attitude that PLs can adopt to guide their engagement with athletes. Based on this principle, PLs may be able to develop strength-based and affirming perspectives to recognize how each unique athlete enriches the group, beyond a deficit understanding of difference and disability. By integrating a strength-based perspective with an overt emphasis on differences, this principle reflects the available literature pertaining to the liberatory and affirmation models of disability (Swain & French, 2000), as well as the common critiques of these models (primarily the latter) that problematize the influence of compulsory able-bodiedness, conformity, and the erasure of disability (McRuer, 2006). The specific focus on differences is powerful to create space for athletes to express their identities (e.g., disability, gender, race) comfortably and in an environment that values differences, which is particularly pertinent to the sport experiences of blind and partially sighted people. Since the experiences of blindness or partial sight are largely embodied, sighted PLs are often unable to identify athletes with differing levels of sight without athletes sharing this information with them (Powis, 2020); however, the process of disclosing and advocating for oneself can be dehumanizing, othering, and tiresome for blind and partially sighted people (Ball, Lieberman, Beach, et al., 2022), as is echoed by other diverse athletes (Maher et al., 2023). Supported by the principle of embrace and respect differences, PLs may be able to foster the six experiential elements of QP for blind and partially sighted athletes by cultivating a safe environment for athletes to express themselves authentically, while, reciprocally, ensuring that PLs themselves receive pertinent information that allows them to support diverse athletes during programming.

In addition to the first and second principles, PLs can engage the third principle, *embedding personalization into programming*, to foster quality sport experiences for blind and partially sighted athletes. This principle reflects the importance of considering the individuality of the person, their needs, and their preferences to foster meaningful and supportive experiences – the absence of which has been suggested as a limitation of person-centred approaches (Parmenter & Arnold, 2008). More specifically, there is a pervasive misconception that people can either see or they cannot, similar to the ableist dichotomies of normal/disabled and normal/abnormal (Healey, 2017; Hill-Briggs et al., 2007). Consequently, this misconception manifests as erroneous assumptions about blind and partially sighted people that erase diversity and distort their preferences, support needs, and expectations in sport contexts (Powis, 2020). As such, the provision of support, tailored to the unique individual, is a particularly powerful method of fostering QP. Moreover, by positioning personalization as a feature of programming that is offered to the entire group, PLs may be able to minimize the othering experienced by blind and partially sighted athletes when they are the sole recipients of support and decrease the tiresome task of navigating existing, inaccessible policies (Ball, Lieberman, Beach, et al., 2022).

Recognizing the role that PLs have in the relational network of people who shape the sport context (Haegele & Maher, 2023), we outline these three principles and 33 distinct strategies through which they may be able to foster QP in sport for blind and partially sighted people. Informed by the unique knowledge in this body of literature constructed by scholars in conversation with blind and partially sighted athletes, the insight we offer in this scoping review represents both novel additions to and reconceptualizations of the foundational strategies represented within the QP framework, which are currently oriented towards the QP of autistic children (Streatch et al., 2022), children with intellectual and developmental disabilities (Bruno et al., 2022), and disabled people more generally (CDPP, 2018). Moreover, we have suggested a broader definition of the physical environment category currently represented in the blueprints (e.g., see CDPP, 2018) to include the multi-sensory aspects of the places and spaces in which sport programming takes place.

## Limitations and future directions

Having critically and extensively reviewed the 29 studies, we developed a unique perspective on the available literature, supporting us in highlighting various limitations of the current body of literature. Of the literature implicated in this review, the influence of intersecting identities on the lived experiences of athletes was largely overlooked. For example, few articles included the voices of older adults, with the oldest participant being 70 years old, and the majority of studies neglected to report the race/ethnicity of participants. However, it may be pertinent to consider the intersecting identities of athletes due to the salience of co-existing identities for the quality of sport experiences (The Sport Information Resource Centre, 2022) and due to the nature of disability as a complex construct with various dimensions including embodied, social, and political (Peers et al., 2014). While intersecting analyses were not prevalent in the reviewed literature, some scholars suggested the exploration of the cultural factors on participation and quality experiences, both to enrich programming and to effectively support diverse participants, as a future direction (e.g., Esatbeyoglu et al., 2023; Hall et al., 2023).

Moreover, the education sector was highly influential on the articles included in this review, with many articles centred on sport and physical education. In particular, the majority of the included articles referenced inclusion, a term that originated from the education sector, as their goal and/or adopted inclusive models of participation; however, the concept of inclusion often remained undefined by the authors – a pattern noted and problematized by Haegele and Maher (2023) due to the varied definitions, uses (e.g., as a programme model, outcome), and ideological underpinnings. In the future, it may be valuable for authors to engage in a reflexive process, as suggested by Haegele and Maher (2023), to clarify their use of the term and ensure that readers’ understandings of inclusion are in alignment with that of the authors. These reflections would have allowed us, in the present review, to further situate QP and subjective in-the-moment experiences in relation to these perspectives on inclusion.

Related to the present review, various limitations influenced our searches of the databases, including the restrictions to English language and peer-review studies. Future reviews would benefit from the inclusion of available grey literature to consider the potential insight represented within informal education (i.e., generated through experiences), grassroots and community-based organizations, and other perspectives not currently represented within mainstream databases. The team engaged in this project included diverse people with expertise in the field of disability and sport, as well as people who experience disability, although the team did not include a blind or partially sighted researcher or community member. Reflecting the participatory and emancipatory methods for which Haegele and Maher (2023) advocated, we will engage with blind and partially sighted community members as we continue to integrate these findings within the larger project and disseminate the generated insight.

## Conclusions

Ultimately, this scoping review contributes to the available literature pertaining to the quality experiences and participation of blind and partially sighted athletes in sport. By engaging with this body of literature, we have integrated the rich insights offered by scholars, in conversation with the perspectives of blind and partially sighted people, into the QP framework to support the development and provision of quality sport programming. In particular, this project provides three unique principles associated with the quality experiences of blind and partially sighted athletes, as well as 33 generated strategies through which these principles are operationalized. By adopting these principles as an ethos to guide their programming, PLs and other supporters may be able to improve the quality of available programming in ways that affirm the identities and experiences of diverse athletes. The insight generated throughout this project will be disseminated to sport programmers and organizations through the creation and publication of a CDPP blueprint (see examples at www.cdpp.ca).

## Supplemental Material

sj-docx-1-jvi-10.1177_02646196251330155 – Supplemental material for The sport experiences of blind or partially sighted people and strategies to support their participation in sport: A scoping review Supplemental material, sj-docx-1-jvi-10.1177_02646196251330155 for The sport experiences of blind or partially sighted people and strategies to support their participation in sport: A scoping review by Meredith K Wing, Julia Deuville, and Alyssa C Grimes, Zachary Scanlan, Kelly Arbour-Nicitopoulos and Amy E Latimer-Cheung in British Journal of Visual Impairment

sj-docx-2-jvi-10.1177_02646196251330155 – Supplemental material for The sport experiences of blind or partially sighted people and strategies to support their participation in sport: A scoping review Supplemental material, sj-docx-2-jvi-10.1177_02646196251330155 for The sport experiences of blind or partially sighted people and strategies to support their participation in sport: A scoping review by Meredith K Wing, Julia Deuville, and Alyssa C Grimes, Zachary Scanlan, Kelly Arbour-Nicitopoulos and Amy E Latimer-Cheung in British Journal of Visual Impairment

## Notes

1. Throughout this article, we thoughtfully employ identity-first language to reflect the influence of blindness and partial sight on the experiences of an individual with the world and to support the advocates who call for the reconstruction of a positive disability identity (Dunn & Andrews, 2015; Peers et al., 2014; Ricker, 2023).
