# Supplementary material for: The sport experiences of blind or partially sighted people and strategies to support their participation in sport: A scoping review
Source: Br J Vis Impair. 2025 May 15;44(2):584–616. doi: 10.1177/02646196251330155 (PMC13189401; doi:10.1177/02646196251330155)
Supplement: sj-docx-1-jvi-10.1177_02646196251330155 – Supplemental material for The sport experiences of blind or partially sighted people and strategies to support their participation in sport: A scoping review [file sj-docx-1-jvi-10.1177_02646196251330155.docx]

**Appendix A**

**Search Strategy**

**ABSTRACT** "visual disab*" OR "vision loss" OR "sight loss" OR "partially sighted" OR "visual impairment*" OR "visually impaired" OR blind OR blindness OR “low vision” OR "vision disorder*" OR goalball

**ABSTRACT** sport OR sports OR “blind sport*” OR paralympics OR para OR athlete* OR tournament OR competition OR “physical training*” OR “sports training” OR “sporting event*” OR “sports camp*” OR “physical activit*” OR athlete* OR athletics

**FULL TEXT** experience* OR perception* OR participation OR participate OR involvement OR involv* OR attitude* OR view* OR perspective* OR qualitative OR “interpersonal relationship*” OR parent* OR “coach-athlete relationship*” OR peer OR “social support” OR barrier* OR facilitat* OR “socioeconomic factor” OR “psychosocial factor*” OR famil* OR support OR coach* OR team* OR trainer* OR teach* OR instruct* OR inclusion OR integrat* OR communit* OR communication OR volunteer* OR strateg* OR “quality participation” OR “positive experience*” Or engage*

**FULL TEXT** **NOT** metabol* OR "body composition" OR postconcuss* OR headache OR “single-blind” OR “double-blind” OR "blind experiment"
