# Supplementary material for: The sport experiences of blind or partially sighted people and strategies to support their participation in sport: A scoping review
Source: Br J Vis Impair. 2025 May 15;44(2):584–616. doi: 10.1177/02646196251330155 (PMC13189401; doi:10.1177/02646196251330155)
Supplement: sj-docx-2-jvi-10.1177_02646196251330155 – Supplemental material for The sport experiences of blind or partially sighted people and strategies to support their participation in sport: A scoping review [file sj-docx-2-jvi-10.1177_02646196251330155.docx]

**Appendix B**

*Included Studies*

Barnett, B. E., Merriman, W. J., & Lupo, S. Q. (1993). Personal values in sport of visually impaired and sighted wrestlers. *Perceptual and Motor Skills*, *77*(3), 816–818. [<https://doi.org/10.2466/pms.1993.77.3.816>](https://doi.org/10.2466/pms.1993.77.3.816)

Braga, L., Tracy, J. F., & Taliaferro, A. R. (2015). Physical activity programs in higher education: Modifying net/wall games to include individuals with disabilities. *Journal of Physical Education, Recreation & Dance*, *86*(1), 16–22. [<https://doi.org/10.1080/07303084.2014.978417>](https://doi.org/10.1080/07303084.2014.978417)

Brunes, A., Krokstad, E., & Berit Augestad, L. (2017). How to succeed? Physical activity for individuals who are blind. *The British Journal of Visual Impairment*, *35*(3), 264–274. [<https://doi.org/10.1177/0264619617716720>](https://doi.org/10.1177/0264619617716720)

Columna, L., Fernández-Vivó, M., Lieberman, L., & Arndt, K. (2015). Recreational physical activity experiences among Guatemalan families with children with visual impairments. *Journal of Physical Activity & Health*, *12*(8), 1119–1127. [<https://doi.org/10.1123/jpah.2014-0257>](https://doi.org/10.1123/jpah.2014-0257)

Columna, L., Rocco Dillon, S., Norris, M. L., Dolphin, M., & McCabe, L. (2017). Parents’ perceptions of physical activity experiences for their families and children with visual impairments. *The British Journal of Visual Impairment*, *35*(2), 88–102. [<https://doi.org/10.1177/0264619617691081>](https://doi.org/10.1177/0264619617691081)

de Schipper, T., Lieberman, L. J., & Moody, B. (2017). “Kids like me, we go lightly on the head”: Experiences of children with a visual impairment on the physical self-concept. *The British Journal of Visual Impairment*, *35*(1), 55–68. [<https://doi.org/10.1177/0264619616678651>](https://doi.org/10.1177/0264619616678651)

Esatbeyoglu, F., Kirk, T., & Haegele, J. A. (2023). “Like I’m flying”: Capoeira dance experiences of youth with visual impairments. *The British Journal of Visual Impairment*, *41*(2), 243–253. [<https://doi.org/10.1177/02646196211059756>](https://doi.org/10.1177/02646196211059756)

Gombás, J., & Gál, A. (2016). The involvement of Budapest residents with visual impairments in leisure sports: Barriers and facilitators. *Physical Culture and Sport Studies and Research*, *70*(1), 44–54. [<https://doi.org/10.1515/pcssr-2016-0008>](https://doi.org/10.1515/pcssr-2016-0008)

Goodwin, D. L., Lieberman, L. J., Johnston, K., & Leo, J. (2011). Connecting through summer camp: Youth with visual impairments find a sense of community. *Adapted Physical Activity Quarterly*, *28*(1), 40–55. [<https://doi.org/10.1123/apaq.28.1.40>](https://doi.org/10.1123/apaq.28.1.40)

Green, C., & Miyahara, M. (2007). Older adults with visual impairment: Lived experiences and a walking group. *RE:View*, *39*(3), 91–112. [<https://doi.org/10.3200/REVU.39.3.91-112>](https://doi.org/10.3200/REVU.39.3.91-112)

Haegele, J. A., Lieberman, L. J., Lepore, M., & Lepore-Stevens, M. (2014). A service delivery model for physical activity in students with visual impairments: Camp Abilities. *Journal of Visual Impairment & Blindness*, *108*(6), 473–483. [<https://doi.org/10.1177/0145482X1410800604>](https://doi.org/10.1177/0145482X1410800604)

Haegele, J. A., Zhu, X., & Davis, S. (2017). The meaning of physical education and sport among elite athletes with visual impairments. *European Physical Education Review*, *23*(4), 375–391. [<https://doi.org/10.1177/1356336X16650122>](https://doi.org/10.1177/1356336X16650122)

Hall, D. L., Allen-Collinson, J., & Jackman, P. C. (2023). “The agenda is to have fun”: Exploring experiences of guided running in visually impaired and guide runners. *Qualitative Research in Sport, Exercise and Health*, *15*(1), 89–103. [<https://doi.org/10.1080/2159676X.2022.2092200>](https://doi.org/10.1080/2159676X.2022.2092200)

Kirk, T. N., & Haegele, J. A. (2021). Expectancy-value beliefs, identity, and physical activity among adults with visual impairments. *Disability and Rehabilitation*, *43*(4), 516–524. [<https://doi.org/10.1080/09638288.2019.1631395>](https://doi.org/10.1080/09638288.2019.1631395)

Laughlin, M. K., & Happel, K. (2016). Developing an appropriate goalball unit for secondary physical education. *Strategies*, *29*(1), 16–23. [<https://doi.org/10.1080/08924562.2015.1111784>](https://doi.org/10.1080/08924562.2015.1111784)

Lepore-Stevens, M., Adams, D., Lepore, M., & Foster, E. A. (2021). Camp Abilities: Accessibility and virtual summer camps. *Journal of Park and Recreation Administration*, *39*(4), 141-151. [<https://doi.org/10.18666/JPRA-2021-10752>](https://doi.org/10.18666/JPRA-2021-10752)

Lieberman, L. J., Robinson, B. L., & Rollheiser, H. (2006). Youth with visual impairments: Experiences in general physical education. *RE:View*, *38*(1), 35–48. [<https://doi.org/10.3200/REVU.38.1.35-48>](https://doi.org/10.3200/REVU.38.1.35-48)

Lieberman, L. J., Schedlin, H., & Pierce, T. (2009). Teaching Jump Rope to Children with Visual Impairments. *Journal of Visual Impairment & Blindness*, *103*(3), 173–178. [<https://doi.org/10.1177/0145482X0910300306>](https://doi.org/10.1177/0145482X0910300306)

Lieberman, L. J., Lepore, M., Lepore-Stevens, M., & Ball, L. (2019a). Physical Education for Children with Visual Impairment or Blindness. *Journal of Physical Education, Recreation & Dance*, *90*(1), 30–38. [<https://doi.org/10.1080/07303084.2018.1535340>](https://doi.org/10.1080/07303084.2018.1535340)

Lieberman, L. J., Haibach-Beach, P. S., Sherwood, J., & Trad, A. (2019b). “We now fly”: Perspectives of adults who are blind with guide dogs trained for running. *The British Journal of Visual Impairment*, *37*(3), 213–226. [<https://doi.org/10.1177/0264619619842989>](https://doi.org/10.1177/0264619619842989)

Macbeth, J. L. (2009). Restrictions of activity in partially sighted football: Experiences of grassroots players. *Leisure Studies*, *28*(4), 455–467. [<https://doi.org/10.1080/02614360903071696>](https://doi.org/10.1080/02614360903071696)

MacDonald, C., Bryan, R., Lieberman, L. J., & Foley, J. T. (2020). “You think differently after playing this sport”: Experiences of collegiate goalball players. *Recreational Sports Journal*, *44*(2), 139–148. [<https://doi.org/10.1177/1558866120964812>](https://doi.org/10.1177/1558866120964812)

Mastro, J. V., Montelione, T. L., & Hall, M. M. (1986). Wrestling: A viable sport for the visually impaired. *Journal of Physical Education, Recreation & Dance*, *57*(9), 61–64. [<https://doi.org/10.1080/07303084.1986.10606199>](https://doi.org/10.1080/07303084.1986.10606199)

Mycock, D., & Molnár, G. (2021). “The blind leading the blind” - A reflection on coaching blind football. *European Journal of Adapted Physical Activity*, *14*(1), 3–3. [<https://doi.org/10.5507/euj.2020.011>](https://doi.org/10.5507/euj.2020.011)

Nixon, H. L. (1988). Getting over the worry hurdle: Parental encouragement and the sports involvement of visually impaired children and youths. *Adapted Physical Activity Quarterly*, *5*(1), 29–43. [<https://doi.org/10.1123/apaq.5.1.29>](https://doi.org/10.1123/apaq.5.1.29)

Nixon, H. L. (1989). Integration of disabled people in mainstream sports: Case study of a partially sighted child. *Adapted Physical Activity Quarterly*, *6*(1), 17–31. [<https://doi.org/10.1123/apaq.6.1.17>](https://doi.org/10.1123/apaq.6.1.17)

Norris, M. L., Toole, K. M., & Columna, L. (2018). Educating parents in aquatics activities for children with visual impairments. *The British Journal of Visual Impairment*, *36*(3), 262–273. [<https://doi.org/10.1177/0264619618784631>](https://doi.org/10.1177/0264619618784631)

O’Connell, M., Lieberman, L. J., & Petersen, S. (2006). The use of tactile modeling and physical guidance as instructional strategies in physical activity for children who are blind. *Journal of Visual Impairment & Blindness*, *100*(8), 471–477. [<https://doi.org/10.1177/0145482X0610000804>](https://doi.org/10.1177/0145482X0610000804)

Ward, S., Farnsworth, C., Babkes-Stellino, M., & Perrett, J. (2011). Parental Influence and the Attraction to Physical Activity for Youths who are Visually Impaired at a Residential–Day School. *Journal of Visual Impairment & Blindness*, *105*(8), 493–498. [<https://doi.org/10.1177/0145482X1110500805>](https://doi.org/10.1177/0145482X1110500805)
